# A model to estimate spleen volume from palpated spleen length in patients with myelofibrosis

**DOI:** 10.1007/s00277-026-07108-8

**Published:** 2026-06-10

**Authors:** Albert Assad, Sue Erickson-Viitanen, J. E. Hamer-Maansson, Evan Braunstein, Peter Langmuir

**Affiliations:** Incyte Corporation, 1801 Augustine Cut-Off, Wilmington, DE 19803 USA

**Keywords:** Myelofibrosis, Splenomegaly, Regression analysis, Clinical trial

## Abstract

Spleen volume is commonly assessed in clinical trials involving patients with myelofibrosis (MF), but direct measurement requires radiologic imaging. In routine clinical settings, spleen length measured by palpation is frequently used as an alternative measure. As the relationship between spleen length and spleen volume is not strictly proportional, interpretation of palpation-based measurements may be variable. In this post hoc analysis, we examined the relationship between palpable spleen length and spleen volume in patients with MF using pooled data from two phase 3 clinical studies. Palpable spleen length and patient weight, height, age, and sex were evaluated using mixed-model regression to identify variables associated with spleen volume. A regression model incorporating palpable spleen length and patient height provided the best fit for predicting spleen volume. The model presented here defines a quantitative relationship between palpable spleen length and spleen volume and provides a standardized reference for relating these measurements in research and observational contexts.

Myelofibrosis (MF) is a myeloproliferative neoplasm characterized by splenomegaly, with 75% to 90% of patients having a palpable spleen at diagnosis [1–3]. Spleen volume has been established as a surrogate measure for disease severity, with reduction in spleen volume an important measure of treatment efficacy that is also used as an endpoint in clinical trials [4]. However, accurate measurement of spleen volume requires radiologic imaging [5], which is not always feasible in a routine clinical setting and cannot be performed regularly. Instead, clinicians often use manual palpation to assess spleen length [5]. Reductions in spleen length have previously been shown to be a reliable corollate for improvements in spleen volume [6]; however, the relationship between spleen volume and spleen length may not be directly proportional. As such, there is a need for a simple but robust method for clinicians to estimate spleen volume based on length measurements, thereby bridging the gap between clinical trial assessments and real-world clinical practice.

In this analysis, we investigated the relationship between spleen volume and palpable spleen length, with considerations for height, weight, age, and sex, in patients with MF from two phase 3 clinical trials to establish and validate a conversion formula between the 2 measurements.

The training and test datasets for the model were based on a post hoc analysis of combined data from 2 double-blind, placebo-controlled phase 3 trials: COMFORT-I (NCT00952289) [4] and LIMBER-313 (NCT04551066). COMFORT-I assessed efficacy and safety of ruxolitinib versus placebo in Janus kinase (JAK) inhibitor–naive patients with International Prognostic Scoring System (IPSS) intermediate-2 or high-risk primary, post–polycythemia vera, or post–essential thrombocythemia MF and palpable spleen at baseline (≥ 5 cm below the left costal margin). Patients had MF that was refractory to available therapies or they were ineligible for available therapies. LIMBER-313 assessed the efficacy and safety of parsaclisib combined with ruxolitinib versus ruxolitinib monotherapy in patients with Dynamic IPSS (DIPSS) intermediate-1 risk or higher primary, post–polycythemia vera, or post–essential thrombocythemia MF and palpable spleen at baseline (≥ 5 cm below the left costal margin) who were naive to JAK or phosphatidylinositol 3-kinase inhibitor treatment. Patients from COMFORT-I and LIMBER-313 were included in this post hoc analysis if they had spleen volume and length measurements at the same timepoint and the spleen length measurement was > 0 cm. COMFORT-I and LIMBER-313 were conducted in accordance with the Declaration of Helsinki and the guidelines for Good Clinical Practice. The protocols were approved by each participating site’s institutional review board. All patients provided written informed consent.

Spleen volume was assessed using magnetic resonance imaging or computed tomography. Palpable spleen length was measured from below the left costal margin to the point of greatest protrusion using a soft ruler. Timepoints included were baseline, Week 12, Week 24, and all subsequent timepoints where both spleen volume and length (> 0 cm) measurements were available. Patient height, weight, age, and sex were captured at baseline for each study.

**Table 1:** Baseline demographics and characteristics of patients in the training and test datasets

Variable	Training dataset	Test dataset
	(*n* = 395 patients)^a^	(*n* = 146 patients)^b^
Median age (range), y	66.0 (32–91)	67.0 (33–86)
Male, n (%)	221 (55.9)	88 (60.3)
Mean weight (SD), kg	72.9 (15.9)	71.3 (14.1)
Mean height (SD), cm	169.4 (9.6)	170.5 (9.6)
Median palpable spleen length (range), cm	12.0 (0–34)^c^	12.0 (5–33)
Median spleen volume (range), cm^3^	1985 (127–8881)	2046 (586–7462)

^a^The training dataset included 1177 pairs of spleen volume and length measurements. ^b^The test dataset included 406 pairs of spleen volume and length measurements. ^c^One patient in the COMFORT-I trial had a baseline spleen length recorded as nonpalpable in error but had a prior measurement of 16 cm and a baseline spleen volume of 2450 cm^3^. The baseline nonpalpable spleen length measurement was not included in the model

Data from the COMFORT-I and LIMBER-313 trials were combined to create a single dataset; 75% of the spleen measurement data were then used as a training dataset to build the model. As spleen measurement data were collected at multiple timepoints, the randomization process was adjusted to ensure all spleen measurements from a given patient were included in either the training or test dataset. A mixed-model regression analysis was used to determine which covariates (palpable spleen length, spleen length squared, height, weight, age, and sex) best predicted spleen volume at each timepoint that spleen volume was collected. There were no adjustments made for repeated measures within the same patient. A postfitting linear model was used to test the regression model with the remaining 25% of the combined spleen measurement data. The mixed-model regression used in this analysis was selected based on the Akaike information criterion (AIC). The AIC balances model fit with model complexity. The model presented in this publication was chosen based on simplicity and acceptable accuracy. SAS v9.4 was used to evaluate all models.

Machine learning approaches, including random forest, support vector machines, boosting, and multi-layer perceptrons, were explored but did not achieve a significant improvement in predictive accuracy. The root mean square error was used to evaluate the machine learning approaches to find the best model.

This post hoc analysis included 541 patients (96.4% of the total combined COMFORT-I and LIMBER-313 population [*N* = 561]). Baseline demographics and characteristics of patients in the training (*n* = 395 [including 1177 pairs of spleen volume and length measurements]) and test (*n* = 146 [including 406 pairs of spleen volume and length measurements]) datasets are shown in Table 1. The regression model that best fit the data for the training and test datasets is as follows:

*V=137L+34H-5227*

*V* represents spleen volume (cm^3^); *L* represents palpable spleen length (cm); *H* represents patient height (cm).

As an example, using the regression model defined here, a palpable spleen length of 10 cm below the costal margin for a patient who is 170 cm tall would equate to a volume of 1923 cm^3^ (95% CI: 257, 3409; Fig. 1). Building on the example, to achieve a 35% reduction in spleen volume (a common clinical trial endpoint), the regression model indicates palpable spleen length would need to decrease to approximately 5.1 cm (corresponding to a spleen volume of 1250 cm^3^).

**Fig. 1:**
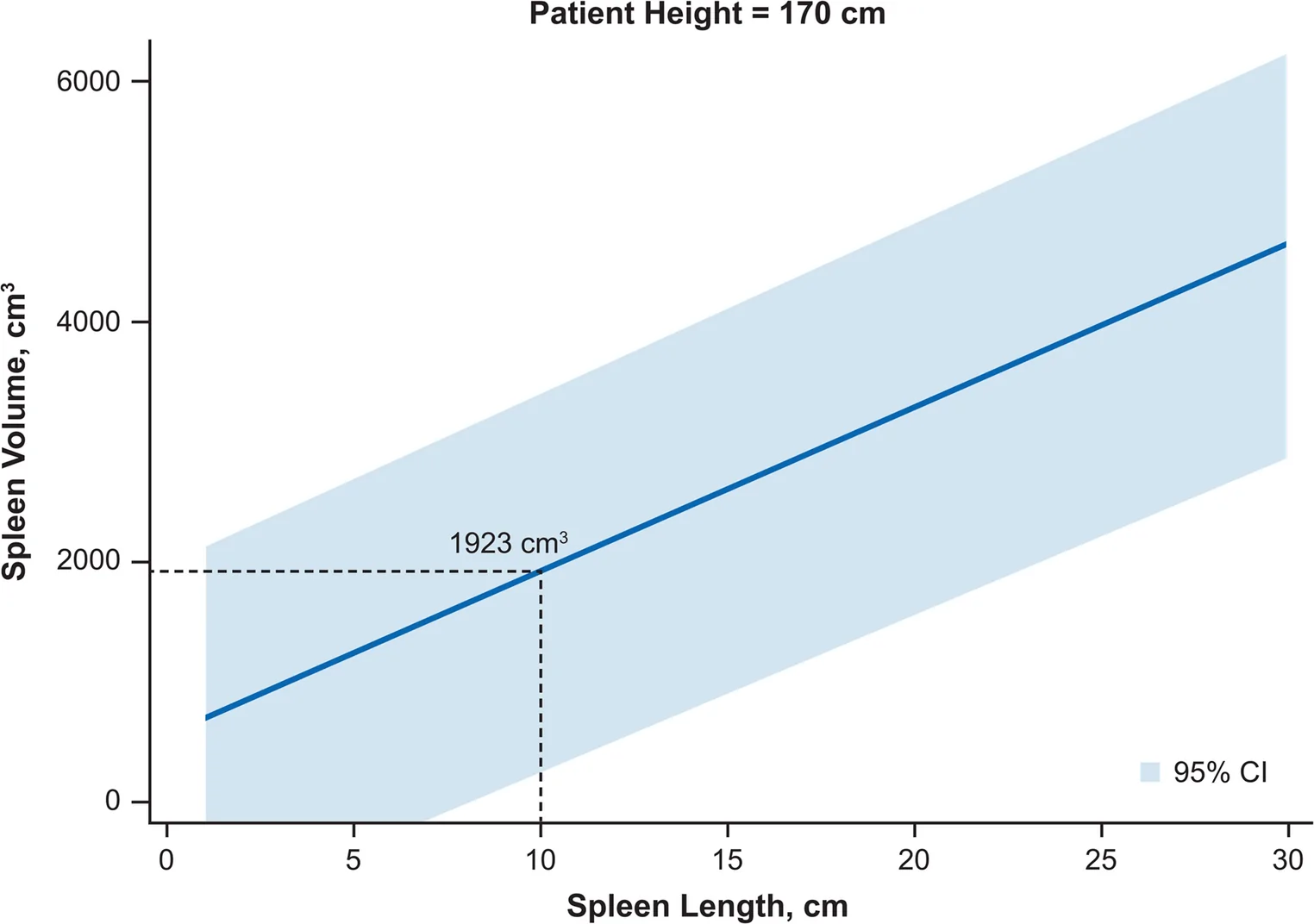
Relationship between spleen volume and palpable spleen length. Graphical representation of the model output, varying spleen volume and length, highlighting an example patient of 170 cm height with a spleen length of 10 cm

This post hoc training and validation analysis of two phase 3 clinical trials established a regression model that predicts spleen volume using spleen length and patient height in patients with MF who have a palpable spleen. This finding has important practical implications, as the tool could be used by clinicians to estimate spleen volume from manual assessments of spleen length in combination with patient height, reducing reliance on time-consuming, costly, and often impractical radiologic imaging assessments.

This analysis is limited by its exploratory post hoc nature and its validation in pooled JAK inhibitor–naive patients with a range of IPSS and DIPSS scores. The regression model should be validated in different patient populations, including those previously treated with JAK inhibitors, and patients categorized by disease severity. The model presented here is also based on MF clinical trial datasets only; future evaluations of the model in real-world datasets and datasets from patients with other myeloproliferative neoplasms such as polycythemia vera and essential thrombocythemia may further support its clinical relevance. The model relies on spleen length determined through palpation; as such, user-based variability in spleen length measurement may affect model-based spleen volume estimates. Moreover, the model may be limited by the absence of adjustments made for repeated measures within the same patient, which may affect real-world applicability for clinicians using the model in the management of patients with MF. Additional limitations include patient or disease-related characteristics not accounted for that may affect the spleen volume–length relationship. Finally, use of this model is limited to patients with a palpable spleen.

In summary, we describe a regression model that characterizes the relationship between palpated spleen length, patient height, and imaging-based spleen volume in patients with MF. The model offers a quantitative approach for relating palpable spleen length and spleen volume measurements that may be informative in clinical research and real-world observational contexts.

## Data Availability

Incyte Corporation (Wilmington, DE, USA) is committed to data sharing that advances science and medicine while protecting patient privacy. Qualified external scientific researchers may request anonymized datasets owned by Incyte for the purpose of conducting legitimate scientific research. Researchers may request anonymized datasets from any interventional study (except Phase 1 studies) for which the product and indication have been approved on or after 1 January 2020 in at least one major market (e.g. US, EU, JPN). Data will be available for request after the primary publication or 2 years after the study has ended. Information on Incyte’s clinical trial data sharing policy and instructions for submitting clinical trial data requests are available at: https://cdn.incyte.com/Archive/clinical-trial-data-sharing.pdf?.
